# Antiviral Effects of ABMA and DABMA against Influenza Virus In Vitro and In Vivo via Regulating the Endolysosomal Pathway and Autophagy

**DOI:** 10.3390/ijms23073940

**Published:** 2022-04-01

**Authors:** Hongtao Liu, Chunlai Jiang, Yu Wu, Min Wu, Jiaxin Wu, Guanshu Zhao, Jie Sun, Xinyu Huang, Jiemin Li, Rui Sheng, Julien Barbier, Jean-Christophe Cintrat, Daniel Gillet, Weiheng Su

**Affiliations:** 1 National Engineering Laboratory for AIDS Vaccine, School of Life Sciences, Jilin University, Changchun 130012, China; liuht17@mails.jlu.edu.cn (H.L.); jiangcl@jlu.edu.cn (C.J.); minwu19@mails.jlu.edu.cn (M.W.); wujiaxin@jlu.edu.cn (J.W.); zhaoguanshu@foxmail.com (G.Z.); sunjie20@mails.jlu.edu.cn (J.S.); hxinyu0331@163.com (X.H.); ljmin11@163.com (J.L.); shengrui20@mails.jlu.edu.cn (R.S.); 2 Key Laboratory for Molecular Enzymology and Engineering of the Ministry of Education, School of Life Sciences, Jilin University, Changchun 130012, China; 3 Département Médicaments et Technologies pour la Santé (DMTS), CEA, INRAE, Université Paris-Saclay, SIMoS, 91191 Gif-sur-Yvette, France; wuyu81@gmail.com (Y.W.); julien.barbier@cea.fr (J.B.); 4 Département Médicaments et Technologies pour la Santé (DMTS), CEA, INRAE, Université Paris-Saclay, SCBM, 91191 Gif-sur-Yvette, France; jean-christophe.cintrat@cea.fr

**Keywords:** influenza virus, ABMA, DABMA, broad-spectrum antivirals, autophagy

## Abstract

Influenza virus is an acute and highly contagious respiratory pathogen that causes great concern to public health and for which there is a need for extensive drug discovery. The small chemical compound ABMA and its analog DABMA, containing an adamantane or a dimethyl-adamantane group, respectively, have been demonstrated to inhibit multiple toxins (diphtheria toxin, *Clostridium difficile* toxin B, *Clostridium sordellii* lethal toxin) and viruses (Ebola, rabies virus, HSV-2) by acting on the host’s vesicle trafficking. Here, we showed that ABMA and DABMA have antiviral effects against both amantadine-sensitive influenza virus subtypes (H1N1 and H3N2), amantadine-resistant subtypes (H3N2), and influenza B virus with EC_50_ values ranging from 2.83 to 7.36 µM (ABMA) and 1.82 to 6.73 µM (DABMA), respectively. ABMA and DABMA inhibited the replication of influenza virus genomic RNA and protein synthesis by interfering with the entry stage of the virus. Molecular docking evaluation together with activity against amantadine-resistant influenza virus strains suggested that ABMA and DABMA were not acting as M2 ion channel blockers. Subsequently, we found that early internalized H1N1 virions were retained in accumulated late endosome compartments after ABMA treatment. Additionally, ABMA disrupted the early stages of the H1N1 life cycle or viral RNA synthesis by interfering with autophagy. ABMA and DABMA protected mice from an intranasal H1N1 challenge with an improved survival rate of 67%. The present study suggests that ABMA and DABMA are potential antiviral leads for the development of a host-directed treatment against influenza virus infection.

## 1. Introduction

Influenza virus is an acute and highly contagious respiratory pathogen, posing a threat to society and public health [1]. The influenza virus is a segmented negative strand RNA virus that belongs to the *Orthomyxoviridae* family. Influenza A viruses (IAV), especially H1N1 and H3N2 subtypes, are highly genetically variable and cause millions of severe cases globally each year [2]. Small molecule drugs are the main approach for treating influenza. These include mainly neuraminidase (NA) inhibitors (zanamivir, oseltamivir phosphate), M2 ion channel blockers (amantadine, rimantadine), and viral RNA synthesis inhibitors (ribavirin, favipiravir, and baloxavir marboxil) [3,4]. However, influenza virus mutates frequently, and both NA and M2 inhibitor-resistant strains have been reported [5]. Moreover, the side effects of ribavirin and favipiravir restrict their clinical use [6]. Drug resistant viruses against baloxavir marboxil have already been detected from clinical trials owing to an I38 mutation of the cap-dependent endonuclease of influenza virus [7].

ABMA was identified by a cell-based high-throughput screening against ricin toxin [8]. We demonstrated that ABMA and its optimized analog DABMA can protect cells against multiple toxins (diphtheria toxin, *Clostridium difficile* toxin B, *Clostridium sordellii* lethal toxin, pseudomonas exotoxin A) and viral infections by rabies, herpes simplex virus type 2 (HSV-2), and Ebola virus [9,10,11]. The mechanism of action involves the delay of virus and toxin trafficking through late endosomes by inhibition of the fusion of late endosomes with lysosomes. This decreases the formation of autolysosomes and reduces the autophagic flux [12].

The endolysosomal system consists of complex, highly dynamic membrane-enclosed tubular-vesicular structures and is widely involved in cell metabolism, hormone secretion, and natural immunity [13]. Simultaneously, the endolysosomal pathway plays a crucial role in virus infection. Stadler et al. [14] found that Amiodarone inhibits late endosomes to suppress SARS-CoV infection. Hernáez et al. [15] localized the capsid and inner envelope proteins of African Swine Fever Virus (ASFV) in early endosomes or macropinosomes and showed that ASFV is transported by the endosomal pathway. Macovei et al. [16] proved that the endosomal vesicles transport the human hepatitis B virus (HBV) viral particles to the replication site and provide an appropriate environment for uncoating and nucleocapsid release of the virus. Drews et al. [17] showed that preventing influenza virus trafficking to late endosomes reduces virus infection.

Autophagy is a physiological process unique to eukaryotic cells that involves lysosomes to degrade intracellular components to maintain the structural stability and function of cells [18,19]. Multiple vesicle structures are involved in this procedure. First, membrane fragments from the endoplasmic reticulum and Golgi vesicles form a cup-shaped structure, the phagophore, and wrap intracellular materials to form a closed double-layer membrane vesicle called the autophagosome. Subsequently, it fuses with late endosomes to form the amphisomes and then further fuses with lysosomes to form autolysosomes that degrade the enclosed components [20]. Autophagy is involved in multiple pathogen infections. Taisne et al. [21] showed that the autophagy machinery participates in the final cytoplasmic wrapping of Human cytomegalovirus (HCMV) viral particles. Corona et al. [22] showed that enterovirus remodels autophagy transport by regulating the host’s SNARE proteins to promote virus replication and release. Wang et al. [23] revealed that alteration of autophagy significantly affects the early stages of the H5N1 life cycle or viral RNA synthesis.

Here, we identified the antiviral activities of ABMA and DABMA against influenza virus. Additionally, the underlying antiviral mechanisms of ABMA against influenza virus were uncovered as inhibition of the endolysosomal pathway and autophagy. This opens the prospect for further development of this family of compounds as host-directed anti-influenza virus treatment.

## 2. Results

### 2.1. ABMA and DABMA Protect Cells against Influenza Virus Infection

**Figure 1:**
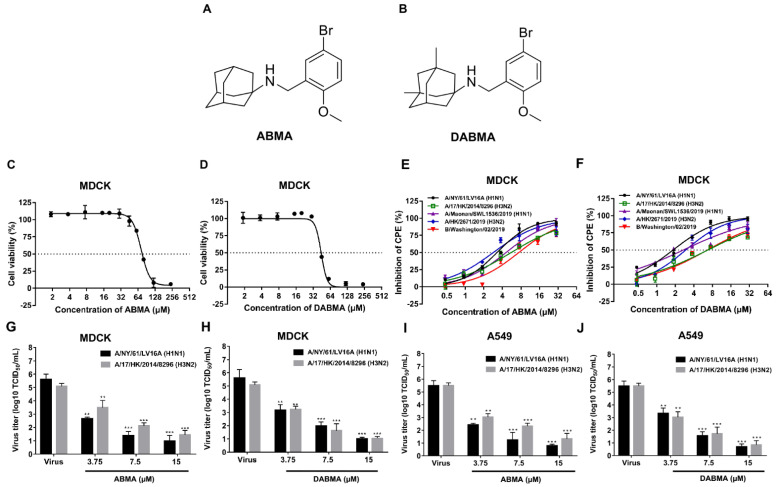
Cytotoxicity of ABMA and DABMA and their antiviral activities against influenza virus in vitro. (**A**,**B**) Chemical structures of ABMA and DABMA. (**C**,**D**) MDCK cells were treated with increasing concentrations of ABMA or DABMA. Cell viability was measured and compared to the untreated cell control after incubation for 72 h. (**E**,**F**) MDCK cells were treated with increasing concentrations of ABMA or DABMA for 5 h before infection with influenza virus (MOI = 0.01). Cytopathic effect was measured and compared to the untreated cell control at 48 hpi. (**G**,**H**) MDCK cells or (**I**,**J**) A549 cells were treated with increasing concentrations of ABMA or DABMA for 5 h before infection with influenza virus (MOI = 0.01). The viral titer (TCID_50_/mL) in supernatants was analyzed at 48 hpi. Data represent mean ± SEM, *n* = 3. The experiments were repeated twice independently. Statistical significance was determined with one way ANOVA. Significance: ** *p* < 0.01, *** *p* < 0.001 compared with virus control group.

We first evaluated the cytotoxicity of the compounds (Figure 1A,B) on MDCK and A549 cells, respectively. The CC_50_ values of ABMA and DABMA were 72.30 ± 1.09 µM and 42.71 ± 1.46 µM in MDCK cells, and were 83.77 ± 1.92 µM and 47.42 ± 1.68 µM in A549 cells, respectively (Figure 1C,D and Table 1).

Structurally, ABMA and DABMA carry an adamantane group (Figure 1A,B). The anti-influenza virus drug amantadine is composed of an adamantane group carrying an -NH2 function. Amantadine exerts its antiviral activity by blocking the viral M2 ion channel, which is required for the acidification of the virus interior after endocytosis. The S31N mutation on the M2 channel reverses the inhibitory effect of amantadine [24]. The antiviral activities of compounds were tested against several contemporary influenza viruses, amantadine sensitive (M2-S31) strains A/NY/61/LV16A (H1N1), A/17/HK/2014/8296 (H3N2) and A/Maonan/SWL1536/2019 (H1N1), and amantadine resistant (M2-S31N) strains A/HK/2671/2019 (H3N2) and IBV, B/Washington/02/2019. ABMA or DABMA showed antiviral activity against all influenza virus strains tested in a dose-dependent manner with EC_50_ values ranging from 2.83 to 7.36 µM (ABMA) and 1.82 to 6.73 µM (DABMA), respectively (Figure 1E,F and Table 1). The positive control drugs oseltamivir, ribavirin, and chloroquine also showed anti-influenza virus activities. Morphological observation of cells showed that treatment with the compounds decreased the cytopathic effect (Figure S1), also proving the protection conferred by the compounds against H1N1 and H3N2 infection. As expected, amantadine did not show antiviral activity against the amantadine resistant (M2-S31N) strains A/HK/2671/2019 (H3N2) and IBV (Table 1), in accordance with previous literature [25]. The inhibitory effects of ABMA and DABMA against amantadine resistant strains indicated that they should have antiviral mechanisms different from amantadine. Moreover, virus titers significantly decreased after treatment with ABMA and DABMA in a dose-dependent manner both in IAV infected MDCK and A549 cells (Figure 1G–J). Altogether, the data indicated that ABMA and DABMA can protect cells against influenza virus infection in vitro in a dose-dependent manner.

### 2.2. ABMA and DABMA Inhibit H1N1 Genomic RNA Replication and Protein Synthesis

**Figure 2:**
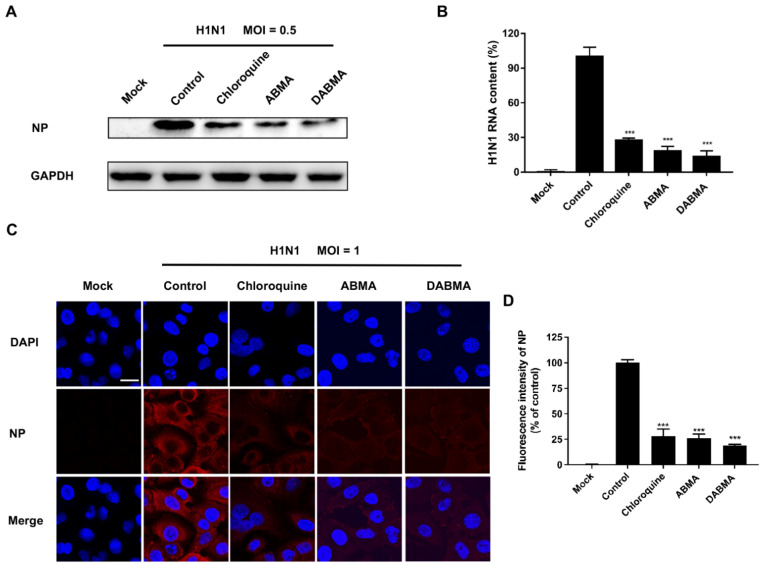
Inhibitory effect of ABMA and DABMA on H1N1 protein synthesis and RNA replication in cell cultures. MDCK cells were pretreated with ABMA or DABMA at 15 μM for 5 h and infected with A/NY/61/LV16A (H1N1, MOI = 0.5) for 1 h at 37 °C. Compounds were maintained until the end of the assay. Chloroquine (15 μM) was used as a positive control. (**A**) Nucleoprotein (NP) was detected by Western blotting using an anti-H1N1 NP antibody and an anti-GAPDH antibody was used as a loading control. (**B**) Relative viral RNA contents were measured by qRT-PCR using glyceraldehyde-3-phosphate dehydrogenase (GAPDH; Proteintech, 60004-1-Ig, Wuhan, China) as an internal control. (**C**) A549 cells were pretreated with ABMA, DABMA, or chloroquine at 15 µM for 5 h. At 16 h post infection with H1N1 (MOI = 1), cells were fixed in 4% paraformaldehyde and incubated with the primary antibody (anti-H1N1 NP, 1:100) overnight at 4 °C and incubated with the Cy5-conjugated goat anti-mouse (red) secondary antibody for 1 h at room temperature. The nuclei were stained with 4′,6-diamidino-2-phenylindole (DAPI; blue). Scale bar: 20 µm. (**D**) Quantitative measurements of fluorescence intensity of NP were evaluated using the Image J software (Version 1.4, National Institutes of Health, Bethesda, MD, USA). Data represent mean ± SEM (*n* = 3). Statistical significance was determined with one way ANOVA. Significance: *** *p* < 0.001 compared with virus control.

To analyze the inhibitory effects of ABMA and DABMA on H1N1 genomic RNA replication and protein synthesis, MDCK cells were pretreated with 15 μM ABMA or DABMA (the concentration of which is approximately the EC_90_ of the compounds, to achieve a strong inhibition) 5 h before virus infection. At 16 hpi, cells were lysed to detect viral nucleoprotein (NP) by Western blotting and RNA copies by qRT-PCR. As shown in Figure 2A, Western blot analysis of NP indicated that expression levels were reduced in compound-treated groups compared to the non-treated controls. qRT-PCR analysis indicated that ABMA and DABMA inhibited 81.77% and 86.51% of H1N1 RNA replication, respectively, which showed significant differences compared with the virus control group (Figure 2B). In addition, H1N1 NP was visualized by immunofluorescence microscopy in the A549 pulmonary cell line. As shown in Figure 2C,D, compared with the virus control group, the fluorescence signal from cells treated with ABMA or DABMA at 15 μM significantly decreased. Collectively, the results demonstrated that ABMA and DABMA inhibit H1N1 RNA replication and protein synthesis in infected cells.

### 2.3. ABMA and DABMA Interfere with the Entry Stage of the H1N1 Infection Cycle

**Figure 3:**
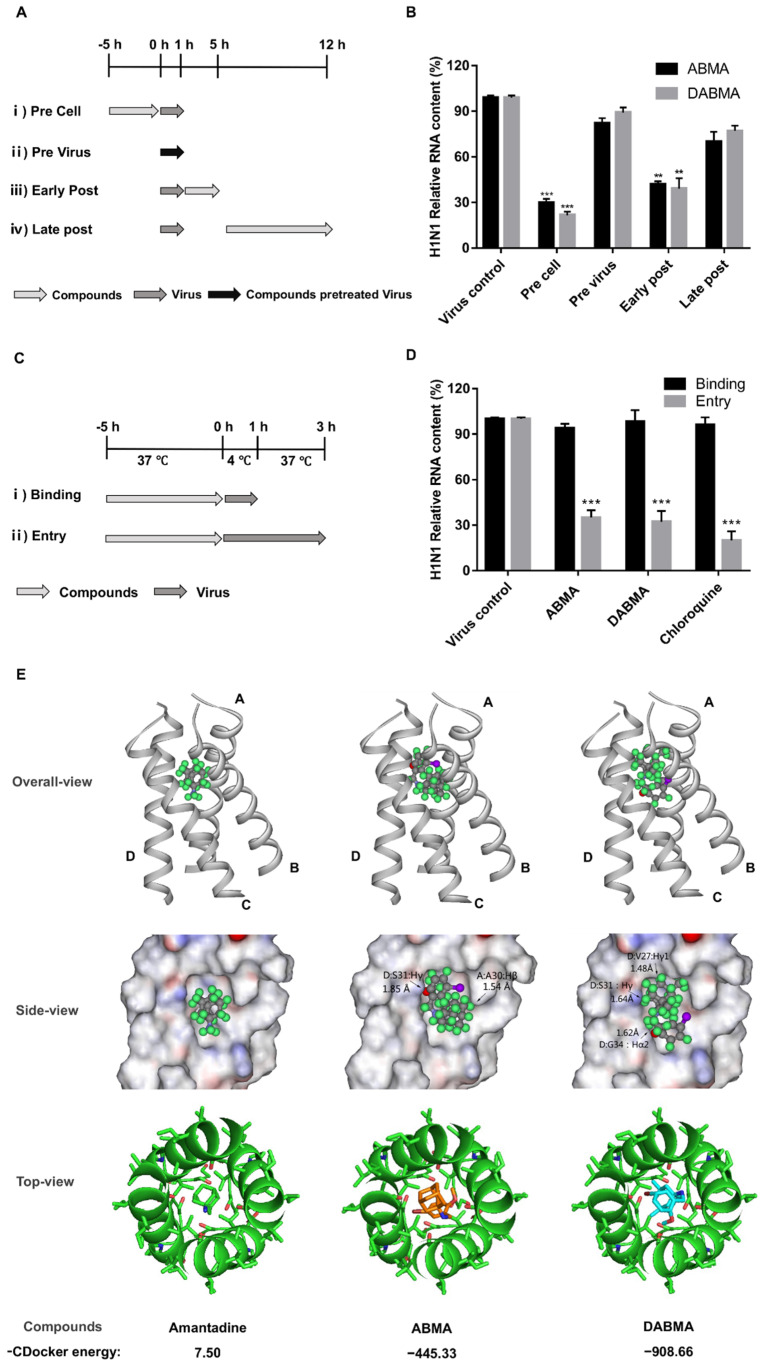
Inhibitory effects of ABMA and DABMA on H1N1 life cycle and M2 channel molecular docking studies. (**A**) ABMA or DABMA treatment of MDCK cells at 15 µM and A/NY/61/LV16A (H1N1, MOI = 1) infection schemes in the time of addition assay. (**B**) Viral RNA was extracted from the cells at 12 hpi and quantified by qRT-PCR. (**C**) ABMA or DABMA treatment of MDCK cells at 15 µM and H1N1 (MOI = 1) infection schemes in the binding and entry assays. (**D**) Viral RNA was extracted from the cell cultures and quantified by qRT-PCR. Data represent mean ± SEM (*n* = 3). The experiments were repeated twice independently. Statistical significance was determined with one way ANOVA. Significance: ** *p* < 0.01, *** *p* < 0.001 as compared to the virus control group. (**E**) Docking models of compounds in the M2 ion channel of IAV. Docking was performed using the crystal structure of the M2-amantadine complex with the PDB 6BKK access number. The −CDocker energies were calculated using Discovery Studio (Accelrys, San Diego, CA, USA). The top panel and bottom panel show the overall and top-view structures of the docking complexes, respectively. The middle panel shows the binding pocket from the side-view, in surface render mode without polypeptide chains B and C of M2. The major clashes of the side chains of the M2 polypeptide chains A and D are labeled by the amino acid residue types and their close inter-molecular distance with the compounds.

To investigate the role of ABMA and DABMA in H1N1 life cycle, time of drug addition experiments were performed in MDCK cells under the schemes shown in Figure 3A. H1N1 RNA content was significantly reduced in pre cell and early post infection treatment groups (Figure 3B). This indicated that ABMA and DABMA impair H1N1 infection at early stages of the virus life cycle. To further explore which process the drugs specifically affect, we conducted binding and entry experiments (Figure 3C,D). As shown in Figure 3D, drug treatment did not affect the binding of the virus to cells. However, the entry stage of H1N1 was significantly inhibited by ABMA and DABMA. This inhibitory effect was similar to that of chloroquine [26,27], which is well known to affect the entry stage of the virus.

ABMA and DABMA showed antiviral activities against amantadine resistant A/HK/2671/2019 (H3N2) strain (M2-S31N) and IBV (Figure 1E,F and Table 1), suggesting that they have a different antiviral mechanism as compared to amantadine. To further rule out this point, the relative binding affinities of amantadine, ABMA, and DABMA with the influenza M2 channel, the target of amantadine, were evaluated using the CDocker molecular docking program in the Discovery studio 2.5 software package. The crystal structure of the M2-amantadine complex (PDB 6BKK) was used for this study. As shown in Figure 3E, the −CDocker energy for ABMA and DABMA was much lower (−445.33 and −908.66, respectively) than that of amantadine (7.50). From the docking results, it seems clear that ABMA binds in a completely different way to M2, in silico, as compared to amantadine. The adamantane moiety of ABMA penetrates deeper within the channel, which results in a steric clash with the side chains of Ser31 in the polypeptide chain D of M2 and Ala30 in the polypeptide chain A of M2 (Figure 3E). Whereas DABMA binds in a more similar orientation as amantadine (“adamantane” stays at the top of the channel), even more steric clashes occur, between DABMA and Val27, Ser31 and Gly34 in polypeptide chain D (Figure 3E). These steric clashes certainly account for the low values of −CDocker energy of ABMA and DABMA with M2. The negative values of the −CDocker energies found for ABMA and DABMA actually suggest that the free energy of the complexes probably increases so that such interactions are unlikely to happen (Figure 3E). Altogether, these results indicated that ABMA and DABMA obstructed the entry stage of H1N1, but not by acting as M2 ion channel blockers.

### 2.4. ABMA Induced Accumulation of H1N1 Virions in Late Endosomes at the Early Stage Post Infection

**Figure 4:**
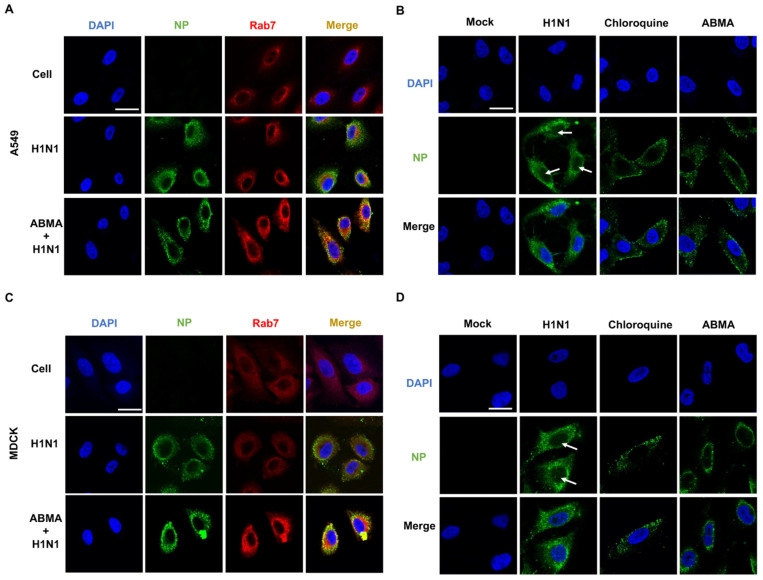
ABMA leads to an accumulation of H1N1 virions in late endosomes. (**A**) A549 cells or (**C**) MDCK cells were pretreated with ABMA (15 µM) for 5 h and infected with A/NY/61/LV16A (H1N1, MOI = 1). At 3 hpi, cells were fixed in 4% paraformaldehyde and incubated with the primary antibodies (mouse monoclonal anti-H1N1-NP, rabbit polyclonal anti-Rab7) overnight at 4 °C and incubated with the FITC-conjugated goat anti-mouse (green) and Cy5-conjugated goat anti-rabbit (red) secondary antibodies for 1 h at room temperature. (**B**) A549 cells or (**D**) MDCK cells were pretreated with ABMA (15 µM) or chloroquine (15 µM) for 5 h and infected with A/NY/61/LV16A (H1N1, MOI = 1). At 8 hpi, cells were fixed in 4% paraformaldehyde and incubated with the primary antibodies (mouse monoclonal anti-H1N1-NP) overnight at 4 °C and incubated with FITC-conjugated goat anti-mouse (green) secondary antibodies for 1 h at room temperature. The nuclei were stained with DAPI (blue). White arrows: NP in the nucleus. Scale bar: 20 µm.

Influenza virus is internalized into cells by endocytosis and transported along the endolysosomal pathway, relying on the acidic environment in the endosomes for fusion of the viral and endosomal membranes, and subsequent uncoating [28]. The viral ribonucleoprotein complexes (RNPs) that are formed of the nucleoproteins (NPs) associated with the viral RNA genome segments are released in the cytoplasm and transported to the cell nucleus. To further explore the mechanism of ABMA inhibition of H1N1 infection, we performed immunofluorescence experiments of H1N1 NPs at 3 and 8 hpi. At 3 hpi, we also detected Rab7, a marker of late endosomes. The Rab7 protein fluorescence was largely increased after ABMA treatment in A549 cells (Figure 4A,C). This indicated that, as expected, ABMA treatment led to an accumulation of late endosomes [8,12]. In addition, the fluorescent labeling of virion NPs increased and co-localized with Rab7. These results indicated that virions accumulated in the late endosomes.

To further explore whether the transportation process of RNPs to the cell nucleus was blocked, we located NPs at the late stage of virus infection (8 hpi). Results showed that in the absence of treatment, green-fluorescent labeling of RNPs was found in the cytoplasm and in the cell nuclei, as expected. In contrast, the fluorescence was mainly distributed outside the nuclei after treatment with the compounds (Figure 4B,D). In addition, the fluorescence seemed to be associated with vesicular compartments in treated cells while in untreated cells it appeared more diffuse. Altogether, these results indicated that virions were accumulated in late endosomes after ABMA and DABMA treatment, hindering RNPs release in the cytoplasm and transport to the nuclei.

### 2.5. ABMA Inhibits H1N1 Replication by Interfering with Autophagy

**Figure 5:**
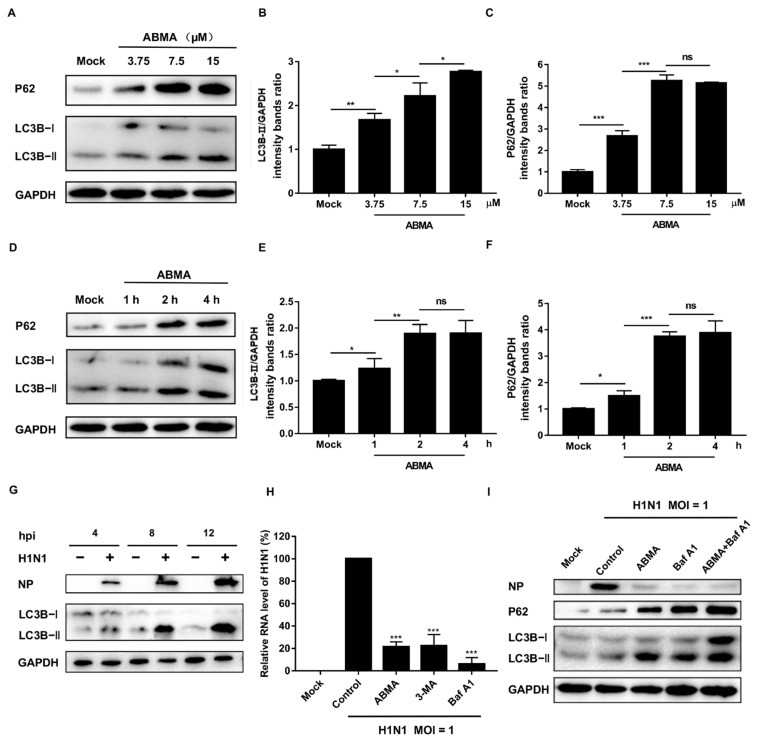
Inhibitory effects of ABMA on the autophagic flux and subsequent impacts on H1N1 infection. (**A**–**C**) A549 cells treated with different concentrations of ABMA were harvested after 12 h of treatment and the autophagy markers LC3B-II and p62 were analyzed using Western blotting. (**D**–**F**) A549 cells treated with 15 µM ABMA were harvested at different time points and the autophagy markers LC3B-II and p62 were analyzed using Western blotting. The band intensities were quantified using Image J software. (**G**) A549 cells were mock infected or infected with A/NY/61/LV16A (H1N1, MOI = 1) at different time points and the autophagy markers were analyzed using Western blotting. (**H**) A549 cells treated with 15 µM ABMA, 5 µM 3-MA, or 100 nM Baf A1 for 5 h were then mock infected or infected with the H1N1 virus (MOI = 1). Total RNA was extracted at 8 hpi, and the mRNA levels of the NP segment were quantified by qRT-PCR. Data represent mean ± SEM (*n* = 3). The experiments were repeated twice independently. Statistical significance was determined with a one way ANOVA. Significance: * *p* < 0.05, ** *p* < 0.01, *** *p* < 0.001, ns represents no significant difference. (**I**) A549 cells treated with 15 µM ABMA, 100 nM Baf A1, or 15 µM ABMA + 100 nM Baf A1 for 5 h were mock infected or infected with the H1N1 virus (MOI = 1). Cell lysates were harvested at 8 hpi and the autophagy markers were analyzed using Western blotting.

Autophagy converges with the endolysosomal pathway through the fusion of autophagosomes with late endosomes to give amphisomes that fuse with lysosomes to form the autolysosomes. In the autolysosomes, the sequestered proteins and dysfunctional cellular components are digested by lysosomal hydrolases [29]. In our previous work, we demonstrated that ABMA modifies the autophagic flux by accumulating amphisomes and inhibiting autolysosome formation [12]. Thus, we hypothesized that ABMA inhibits H1N1 replication by acting on autophagy. To identify the effects of ABMA on autophagy, we treated A549 cells with different concentrations of ABMA and then detected the expression of microtubule-associated protein 1 light chain 3 (LC3), which is widely considered as the marker of autophagosomes. LC3B-I is modified into the phosphatidylethanolamine-conjugated form LC3B-II, which specifically binds to autophagosome membranes when autophagy is induced [30]. LC3B-II was increased in the ABMA-treated cells in a dose- and time-dependent manner (Figure 5A,B,D,E), indicating the accumulation of autophagosomes after ABMA treatment. However, the increased LC3B-II levels may be the result of the activation of autophagy, or alternatively, the inhibition of autophagic degradation. Thus, we monitored the level of p62 in parallel and found the amount of autophagy substrate p62 increased in the ABMA-treated cells in a dose- and time-dependent manner (Figure 5A,C,D,F). Increased levels of LC3B-II and p62 are typically indicative of a defective autophagic flux [31]. This suggested that the ABMA-induced accumulation of autophagosomes was due to inhibition of autophagy.

It was reported that inhibition of autophagy disrupts the early stages of IAV life cycle or viral RNA synthesis [23]. To examine the effects of H1N1 infection on autophagy, A549 cells infected with H1N1 were harvested at different times post infection and tested for the level of LC3B-II by Western blotting. Cells infected with H1N1 displayed an increased expression of LC3B-II in a time-dependent manner (Figure 5G), indicating that H1N1 infection causes the accumulation of autophagosomes. To identify the effect of autophagy on H1N1 infection, we inhibited autophagy by Baf A1 and 3-MA and quantified the H1N1 RNA level [32,33]. As shown in Figure 5H, H1N1 RNA content was significantly decreased after treatment with autophagy inhibitors, indicating that the throttling of autophagy limits H1N1 infection. Additionally, we tested the influence of autophagy on H1N1 protein synthesis by Western blotting. The results showed that the expression levels of NP were decreased after treatment with ABMA or Baf A1, indicating that viral translation was inhibited (Figure 5I). In summary, the results suggest that ABMA affects the early stages of the viral life cycle or viral RNA synthesis via impairing autophagy.

### 2.6. ABMA and DABMA Protect Mice from H1N1 Infection

**Figure 6:**
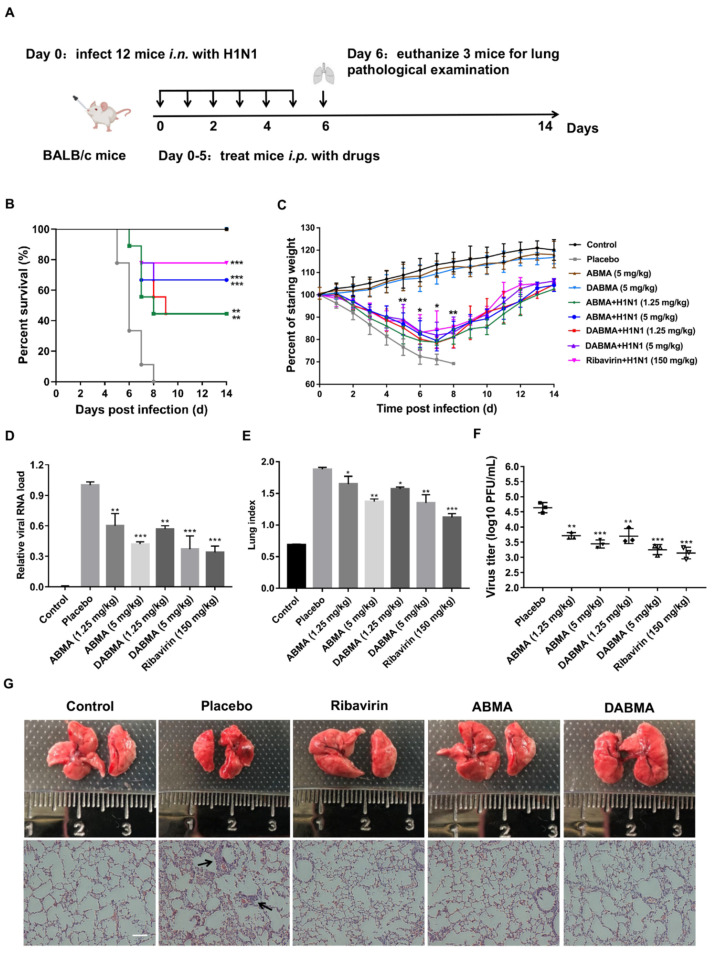
Evaluation of the therapeutic efficacy of ABMA and DABMA in H1N1 inoculated mice. (**A**) BALB/c mice were mock-infected or infected with H1N1 (1 × 10^9^ EID_50_/50 µL) and were injected intraperitoneally with the different compounds once a day for six consecutive days. Survival rate (**B**) and body weight (**C**) of the mice (*n* = 9) were recorded daily for 14 days. Three additional mice in each group were sacrificed at 6 dpi for lung viral load (**D**), lung index (**E**), lung virus titer (**F**) determination, and lung morphological and H&E staining histopathological examinations (**G**). Data represent mean ± SEM. The experiments were repeated twice independently. Statistical significance was determined with one way ANOVA. Significance: * *p* < 0.05, ** *p* < 0.01, *** *p* < 0.001 compared with the placebo group in (**B**,**D**–**F**). Significance: * *p* < 0.05, ** *p* < 0.01 for ABMA + H1N1 (5 mg/kg) or DABMA + H1N1 (5 mg/kg) compared with the placebo group in (**C**). Scale bar: 100 µm. Black arrows indicate inflammatory cell infiltration.

To investigate the antiviral activities of ABMA and DABMA in vivo, mice were inoculated with H1N1 intranasally (i.n.) and were injected with the compounds intraperitoneally (i.p.) (Figure 6A). The mice with >25% body weight loss were euthanized and considered as dead from the infection [34]. As shown in Figure 6B, at a treatment dose of 5 mg/kg of ABMA or DABMA, mice were protected from H1N1 challenge with an improved survival rate of 67% compared with the placebo group. No mice survived in the placebo group beyond day 8. No remarkable changes were observed in animal behavior, lethargy, and body weight in uninfected mice treated at the dose of 5 mg/kg of ABMA or DABMA (Figure 6C), suggesting that ABMA and DABMA are nontoxic at the doses administered. The body weight of mice in the placebo group decreased significantly and all mice reached 75% of their initial weight between days 5 and 8 (Figure 6C). In contrast, the mice in the drug-administered groups rose steadily from day 7 and the difference in body weight was statistically significant between the ABMA (5 mg/kg) or DABMA (5 mg/kg) treatment and placebo group on days 5–8 (Figure 6C). Three mice per group were sacrificed for lung macroscopic and histopathologic examination at 6 dpi (Figure 6A). ABMA and DABMA treatment significantly reduced the lung viral load and lung index compared to the placebo, demonstrating that ABMA and DABMA inhibit viral replication in mouse lungs (Figure 6D,E). Concurrently, lower titers of H1N1 virus were detected in the lungs of ABMA- and DABMA-treated mice, compared to placebo-treated mice (Figure 6F). In addition, H&E staining was used to determine the lung pathology. ABMA and DABMA treatment also reduced the lesions of influenza-induced lung pathology. As shown in Figure 6G, thickening of the pulmonary septum and infiltration of inflammatory cells were observed in the virus-infected mice. However, the lungs of mice treated with ABMA and DABMA showed significant reduction in histopathology changes. These results indicated that ABMA and DABMA could protect mice from the harmful effects of H1N1 infection.

## 3. Discussion

Due to the high public health impact of influenza virus infections, there is a real need for antiviral drugs against this virus. ABMA and its analog DABMA have previously been shown to protect cells against multiple toxins and viruses by interfering with the autophagy–lysosomal pathway [8,9,10]. However, no data are available regarding their antiviral effect against influenza virus.

In this study, the antiviral activities of ABMA and DABMA against influenza virus in vitro were identified for the first time through the virus-induced CPE inhibition experiments. ABMA or DABMA showed antiviral activities against all five tested influenza virus strains in a dose-dependent manner with EC_50_ values ranging from 2.83 to 7.36 µM (ABMA) and 1.82 to 6.73 µM (ABMA), respectively (Figure 1E,F and Table 1). Moreover, ABMA and DABMA were found to effectively inhibit viral NP synthesis and viral RNA replication as shown by Western blotting, qRT-PCR, and immunofluorescence (Figure 2). Therefore, ABMA and DABMA are effective antiviral agents against influenza virus infection in vitro. Subsequently, the antiviral mechanisms of ABMA against H1N1 were further explored. The time of drug addition assay showed that ABMA and DABMA mainly interfere with the early stage of H1N1 infection. Binding and entry experiments proved that ABMA and DABMA do not affect the virus binding but affect its entry stage (Figure 3).

ABMA and DABMA carry an adamantane (ABMA) or dimethyl-adamantane (DABMA) group. Therefore, it must be ruled out whether the antiviral mechanism of these molecules is the same as that of amantadine or not. In terms of structure, ABMA and DABMA are different from amantadine with an additional di-substituted phenyl linked to the adamantane group by an amine linker. In our previous work, we found that amantadine, memantine, and 1-(1-adamantyl) ethylamine (three adamantane analogues with antiviral properties) were inactive against *Corynebacterium diphtheriae*, Ebola virus, and HSV-2 infection, the pathogens of which were inhibited by ABMA and DABMA [8]. Moreover, amantadine provided no significant protection against amantadine-resistant H3N2 strain A/HK/2671/2019 (M2-S31N) and B/Washington/02/2019. On the contrary, ABMA and DABMA showed antiviral activities against these two strains (Table 1). IAV’s M2 ion channel is the target of amantadine. Molecular docking analysis found a much lower −CDocker energy for ABMA and DABMA bound to the M2 ion channel (−445.33 and −908.66, respectively), compared with that of amantadine (7.50), and obvious steric clashes as well (Figure 3E). This suggests that the substituted phenyl group of ABMA and DABMA hinders the binding of the adamantane part to M2. These results indicated that ABMA and DABMA obstructed the entry stage of H1N1, but not by an amantadine-like mechanism that involves blocking of the M2 ion channel.

Influenza viruses are internalized into cells by endocytosis and transported along the endolysosomal pathway, relying on the acidic environment in the endosomes for uncoating [17]. In our previous work, we found that ABMA interferes with the endolysosomal pathway causing the accumulation of late endosomes. This selectively retains epidermal growth factor (EGF) and diphtheria toxin within late endosomes and thus delays their intracellular trafficking [8]. Late endosomes are transfer stations for sorting proteins and lipids and are responsible for the transportation of pathogens [35]. Indeed, we showed co-localization of H1N1 virions and late endosomes using immunofluorescence experiments. Results showed that ABMA accumulated late endosomes and retained the virions in these compartments. Further, we localized NP at the late stage of virus infection. Results showed that the transport of RNPs to the nucleus was blocked (Figure 4).

**Figure 7:**
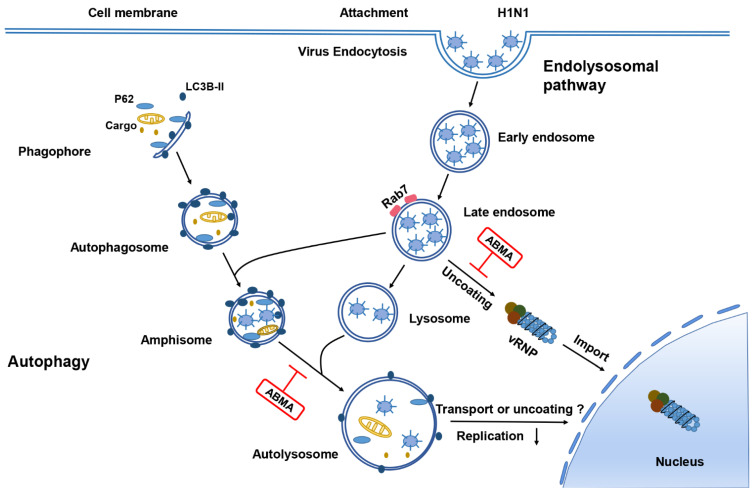
Model depicting the role of ABMA at the early stage of H1N1 infection via the regulation of the endolysosomal pathway and autophagy in mammalian cells.

During viral infection, the virus–host autophagy interaction plays a critical role in the host response to the infection and the viral pathogenicity. Autophagy inhibits pathogenic infection by wrapping viruses in the autophagosome. However, much evidence indicates that virus-induced autophagy plays an important role in the viral life cycle and pathogenicity. The membranes of autophagy compartments can serve as a platform for viral genome replication and transport, such as Coxsackievirus B3, Human cytomegalovirus (HCMV), and Varicella-Zoster virus (VZV) [21,36,37]. We, thus, further investigated whether ABMA inhibits influenza infection by affecting autophagy. Western blot results showed that ABMA blocked the autophagy flux and caused autophagosome accumulation. The synthesis and replication of H1N1 were inhibited when autophagy was downregulated by the inhibitors (Figure 5). This suggests that the trafficking of influenza virus particles was delayed together with the autophagic vesicles and/or that the uncoating stage of the virus in these acidic vesicles was altered (Figure 7).

ABMA and DABMA at the dose of 5 mg/kg protected 67% of IAV-infected mice compared with the placebo group for which 100% mortality was observed. Meanwhile, lung viral load, lung index, and the lesions induced by IAV were significantly reduced in the drug-treated groups compared to the placebo group. This indicates that ABMA and DABMA have effective anti-influenza activities in vivo (Figure 6).

ABMA targeted the autophagy–lysosomal pathway of the host cells instead of the virus itself. Targeting the host could avert or delay the development of drug resistance that occurs under monotherapy or bitherapy with drugs targeting different lifecycle stages of the virus. Not only may the ABMA/DABMA chemical family be considered for further molecular development in the search for higher antiviral activities, but ABMA and DABMA may be used as new molecular tools to investigate the interplay of influenza virus with the endolysosomal system and autophagy.

In conclusion, we demonstrated the anti-influenza virus activities of ABMA and DABMA for the first time, and further explored the mechanisms of action of ABMA. Therefore, ABMA and DABMA have an interesting potential as the basis for the development of antiviral drugs for the treatment of influenza virus infections.

## 4. Materials and Methods

### 4.1. Reagents, Cells, Viruses, and Mice

ABMA [1-adamantyl (5-bromo-2-methoxybenzyl) amine] and DABMA [1, 3-dimethyl-1-adamantyl (5-bromo-2-methoxybenzyl) amine] (Figure 1A,B) were synthesized in-house. Chloroquine and ribavirin were purchased from Meilun Biotech (Dalian, China). Oseltamivir phosphate, 3-methyladenine (3-MA), and Bafilomycin A1 (Baf A1) were from MCE (Shanghai, China). Amantadine hydrochloride was obtained from Sigma-Aldrich (Saint Louis, MO, USA). The purities of the compounds were higher than 98%, as determined by HPLC. All substances were dissolved in DMSO as stock solutions and diluted to final working solutions as indicated in experiments.

MDCK and A549 cell lines were purchased from ATCC (Manassas, VA, USA). Cells were cultured in DMEM, supplemented with 10% FBS, 100 µg/mL streptomycin, and 100 U/mL of penicillin, and maintained in a 5% CO_2_ atmosphere at 37 °C.

Attenuated reassortant influenza virus strains A/NY/61/LV16A (H1N1), A/17/HK/2014/8296 (H3N2) and wild-type viruses strains A/Maonan/SWL1536/2019 (H1N1), A/HK/2671/2019 (H3N2), and B/Washington/02/2019 were propagated in 9-day-old specific pathogen-free (SPF) chicken eggs [38]. The growth of the influenza virus strains in MDCK cells was measured using infectious virus titer (Figure S2). Viruses were titered by TCID_50_ (50% tissue culture infectious dose) and EID_50_ (50% egg infectious dose) and stored at −80 °C. Influenza virus infection experiments were performed in a biosafety level 2 (BSL-2) laboratory.

The SPF female BALB/c mice (6–8 weeks old) were purchased from the Changchun Institute of Biological Products (Changchun, China) and maintained under SPF conditions with a 12/12 h light/dark cycle. Mouse lethal dose 50% titer (MLD_50_) was calculated to be 1 × 10^7.28^ EID_50_/50 μL using the method of Reed and Muench (Table S1).

### 4.2. Cytopathic Effect (CPE) Inhibition Assay on IAV Infection

Cytotoxicity of compounds in MDCK and A549 cells was measured with CellTiter-Glo^®^ Luminescent Cell Viability Assay (Promega, Madison, WI, USA) and quantified using the PerkinElmer VICTOR™ X2 (Waltham, MA, USA) as reported [39].

The anti-influenza virus activities were evaluated using the CPE inhibition assay. Briefly, MDCK cells were seeded in 96-well plates at a density of 5 × 10^3^ cells/well for 24 h and treated with the indicated concentrations of compounds for 5 h before influenza virus (MOI = 0.01) infection. Then, the cells were infected with H1N1 for 1 h and were cultured at 37 °C in a maintenance medium (DMEM-2% FBS containing 2 µg/mL TPCK-trypsin) and treated with 0.2% DMSO (a vehicle to dissolve test compounds) or compounds at the indicated concentrations. The cytopathic effect was measured with CellTiter-Glo^®^ Luminescent Cell Viability Assay (Promega, Madison, WI, USA) and compared to the untreated cell control at 48 hpi.

The inhibition rate was calculated as follows: inhibition rate (%) = [(A − B)/(C − B)] × 100, where A: mean optical density of test; B: mean optical density of virus controls; C: mean optical density of cell controls. The 50% cytotoxicity concentration (CC_50_) and 50% effective concentration (EC_50_) of the compounds were analyzed using regression analysis.

### 4.3. Quantitative Reverse Transcription PCR (qRT-PCR)

Total RNA was extracted from A/NY/61/LV16A (H1N1) infected MDCK cells using an EasyPure^®^ RNA Kit (TransGen Biotech, Beijing, China). The qRT-PCR was conducted using the One-Step SYBR PrimeScript RT-PCR Kit (Takara Bio, Otsu, Japan) in a Bio-Rad CFX96 system (Hercules, CA, USA). The primer pairs for H1N1 NP and GAPDH were listed as follows: NP-F: 5′-AACTCAGTGATTATGAGG-3′; NP-R: 5′-GAGAATGTTGCACATTCT-3′; GAPDH-F: 5′-CACTCACGGCAAATTCAACGGCAC-3′; GAPDH-R: 5′-GACTCCACGACATACTCAGCAC-3′. The relative copies of H1N1 NP mRNA were determined using the comparative 2^−ΔΔCt^ method, as previously described [40].

### 4.4. Western Blotting Assay

Cells cultured in 6-well plates were lysed using RIPA lysis buffer (Beyotime, P0013B, Shanghai, China). Samples were separated by SDS-PAGE and transferred to a nitrocellulose membrane. The membrane was blocked with 6% non-fat milk for 1 h and incubated at 4 °C overnight with appropriate primary antibodies: anti-NP (Abcam, Ab20343, Cambridge, UK), and anti-GAPDH (Proteintech, 60004-1-Ig, Wuhan, China), anti-LC3B (Cell Signaling Technology, 2775S, Danvers, MA, USA), anti-SQSTM1/P62 (Antibodies-online, ABIN2854836, Aachen, Germany). Then, the membrane was washed and incubated with HRP-labeled secondary antibody at room temperature for 1 h for subsequent detection using enhanced chemiluminescence. The protein band densities were quantified using Image J, normalizing to GAPDH.

### 4.5. Time of Addition Assay

MDCK cells were seeded in 24-well plates to 90% confluence and were infected with A/NY/61/LV16A (H1N1, MOI = 1) under the following conditions: (i) Pre cell: cells were pretreated with 15 µM of ABMA or DABMA for 5 h before H1N1 infection. After 1 h of virus adsorption, the supernatant was replaced with compound-free medium. (ii) Pre virus: ABMA or DABMA (15 µM) pretreated H1N1 was added to MDCK cells and incubated at 37 °C for 1 h; after adsorption, the supernatant was removed, and the cells were overlaid with compound-free medium. (iii) Early post: cells were infected with H1N1 at 37 °C for 1 h, then the supernatant was removed, and cells were overlaid with ABMA- or DABMA-containing medium for 5 h; then, the supernatant was replaced with compound-free medium. (iv) Late post: cells were infected with H1N1 at 37 °C for 1 h; then the supernatant was removed, and cells were overlaid with compound-free medium for 5 h. After that, the supernatant was removed, and drug-containing medium was added. Virus yields in each group were determined by qRT-PCR at 12 h post infection (hpi).

Binding and entry assays were performed as reported previously [9]. In the binding assay, MDCK cells were pretreated with ABMA or DABMA for 5 h. Subsequently, cells were incubated with the virus at 4 °C for 1 h, RNA was extracted from the original virus inoculum and unbound virus supernatant, then was quantified by qRT-PCR to calculate the amount of H1N1 bound to the cells in the binding assay; in the entry assay, cells were incubated with the virus for 2 h at 37 °C after binding, washed, and the internalized virus was recovered by two freeze–thaw cycles. Virus yields were determined by qRT-PCR.

### 4.6. Immunofluorescence Assay

Cells in 24-well plates were pretreated with ABMA, DABMA, or chloroquine for 5 h, respectively, followed by exposure to A/NY/61/LV16A (H1N1, MOI = 1) for 1 h. Unbound virus was removed by washing twice with sterile PBS buffer (Beyotime, C0221A, Shanghai, China). At 3 hpi, the cells were fixed with 4% paraformaldehyde for 20 min and permeabilized with 0.5% Triton X-100 (Sigma-Aldrich, X100, Saint Louis, MO, USA)) diluted in PBS for 10 min, followed by blocking with 1% bovine serum albumin (BSA) at 37 °C for 30 min. Subsequently, the cells were incubated with an anti-H1N1 NP mouse monoclonal antibody and Rab7 rabbit polyclonal antibody (Abcam, ab137029, Cambridge, UK) at a dilution of 1:100 in PBS, respectively, at 4 °C overnight. Cells were then incubated in Cy5-conjugated goat anti-rabbit IgG and FITC-conjugated goat anti-mouse IgG secondary antibodies (Bioss, Beijing, China) at 37 °C for 1 h. Nuclei were stained with DAPI (KeyGEN, Nanjing, China) for 10 min. Images were taken using the Carl Zeiss LSM 710 confocal microscope (Zeiss LSM 710, Jena, Germany) with a 40× objective and analyzed with Zen 2009 Light Edition software (Carl Zeiss, Oberkochen, Germany). Quantitative measurements of fluorescence intensity of 200 cells per group were evaluated using the Image J software (Version 1.4, National Institutes of Health, Bethesda, MD, USA).

### 4.7. Molecular Docking

The relative binding affinity of amantadine, ABMA, and DABMA with the Influenza M2 channel was evaluated using the CDocker molecular docking program in Discovery studio 2.5 software package (Accelrys, San Diego, CA, USA). The crystal structure of the Influenza M2 transmembrane domain bound to amantadine (PDB: 6 BKK) was used to model the interactions [41]. Binding site sphere was defined from the volume of the bound structure of amantadine in the PDB file, with a 5 Å radius. CDocker was run with following parameters: Random conformations and orientations were both set to “20”, the simulated annealing was set to “true”, “CHARMM forcefield” and “grid-based potential” were selected, the Top Hits was set to default value as “10”. After docking runs, the −CDocker Energy was interpreted as an approximated indicator of the binding affinity.

### 4.8. Antiviral Activity of Compounds in H1N1-Inoculated Mice

The 6–8-week-old female BALB/c mice (*n* = 12 per group) were randomly assigned. After anesthesia with diethyl ether, mice were intranasally (i.n.) inoculated with 50 μL of virus suspension containing 1 × 10^9^ EID_50_ of A/NY/61/LV16A (H1N1) (52-fold MLD_50_). Four hours after inoculation, mice were injected intraperitoneally (i.p.) with 50 µL of 150 mg/kg of ribavirin (positive control), 1.25 or 5 mg/kg ABMA, 1.25 or 5 mg/kg DABMA or PBS. The drugs were administered once daily for 6 consecutive days. Nine mice in each group were assigned to monitor the body weight and survival for 14 days (the mice with >25% body weight loss were sacrificed and considered as dead from the infection) [34]. To determine the virus load in organs, 3 mice in each group were assigned to sacrifice on day 6 post inoculation and the lungs were weighed, photographed, homogenized, and clarified by centrifugation. The lung index was calculated using the equation: lung index = lung weight/body weight × 100. The supernatant was assayed for virus load of H1N1 by qRT-PCR assay and titered by plaque assay. To determine the damage in organs, the histopathological analysis was performed using H&E staining on sections of the lung samples. H&E staining was performed according to standard H&E protocol.

### 4.9. Statistical Analysis

All experiments were conducted in triplicates. Graphing and analysis were performed using GraphPad Prism v7 and Image J. Statistical analysis was performed by one-way analysis of variance (ANOVA) test. Data represent mean ± SEM (*n* = 3). Statistical significance was represented by asterisks and was marked correspondingly in the figures: ns; not significant, * *p* < 0.05, ** *p* < 0.01, *** *p* < 0.001.

## Figures and Tables

**Table 1:** Cytotoxicity and antiviral activity of compounds against influenza virus infection.

Compounds	EC_50_ ± SD (μM) ^a^					CC_50_ ± SD (μM) ^b^	
	A/NY/61/LV16A (H1N1)	A/17/HK/2014/8296 (H3N2)	A/Maonan/SWL1536/2019 (H1N1)	A/HK/2671/2019 (H3N2)	B/Washington/02/2019	MDCK	A549
	A/M2-S31	A/M2-S31	A/M2-S31	A/M2-S31N	B/M2		
ABMA	3.25 ± 0.17	5.58 ± 0.38	4.57 ± 0.33	2.83 ± 0.26	7.36 ± 0.65	72.30 ± 1.09	83.77 ± 1.92
DABMA	1.82 ± 0.11	6.73 ± 0.73	2.69 ± 0.26	2.95 ± 0.27	6.62 ± 0.46	42.71 ± 1.46	47.42 ± 1.68
Chloroquine	17.07 ± 2.27	11.97 ± 1.26	ND	ND	ND	76.71 ± 5.08	ND
Oseltamivir	7.97 ± 0.85	7.30 ± 0.79	2.95 ± 0.21	1.79 ± 0.64	8.53 ± 1.64	1893 ± 71.8	ND
Amantadine	9.97 ± 1.34	8.27 ± 0.66	20.19 ± 1.36	>100	>100	476.7 ± 39.49	ND
Ribavirin	1.61 ± 0.62	0.13 ± 0.04	3.20 ± 0.34	19.76 ± 3.26	3.02 ±0.39	5484 ± 255.10	ND

^a^ Concentration of drug that improves the viability of influenza virus-infected MDCK cells by 50%. ^b^ Concentration of drug that reduces the viability of uninfected MDCK or A549 cells by 50%. ABMA: [1-adamantyl (5-bromo-2-methoxybenzyl) amine]; DABMA [1, 3-dimethyl-1-adamantyl (5-bromo-2-methoxybenzyl) amine]; MDCK: Madin-Darby canine kidney cell lines; HK: Hong Kong; NY: New York; ND: not determined.

## Data Availability

The data that support the findings of this study are available from the corresponding author upon reasonable request.

## Supplementary Materials

The following are available online at: https://www.mdpi.com/article/10.3390/ijms23073940/s1.
