# Supplementary material for: Antiviral Effects of ABMA and DABMA against Influenza Virus In Vitro and In Vivo via Regulating the Endolysosomal Pathway and Autophagy
Source: Int J Mol Sci. 2022 Apr 1;23(7):3940. doi: 10.3390/ijms23073940 (PMC8999625; doi:10.3390/ijms23073940)
Supplement: Supplementary file 1 [file ijms-23-03940-s001.zip › ijms-1643988-supplementary.pdf]

Table S1. Determination of the MLD_{50} of influenza virus subtype A/NY/61/LV16A in BALB/c mice. Mice were inoculated intranasally with serial 10-fold dilutions of the virus solution. Body weight of mice were monitored continuously for 14 days (the mice with >25% body weight loss were euthanized and considered dead), and mouse lethal dose 50% (MLD_{50}) titers were calculated using the method of Reed and Muench ($MLD_{50} = 1 \times 10^{7.28}$ EID_{50}/50 μL).

Dilution (EID_{50}/50 μL)	Deaths	Lives	Deaths/Total
1×10^9	10	0	1
1×10^8	8	2	0.8
1×10^7	4	6	0.4
1×10^6	0	10	0
1×10^5	0	10	0

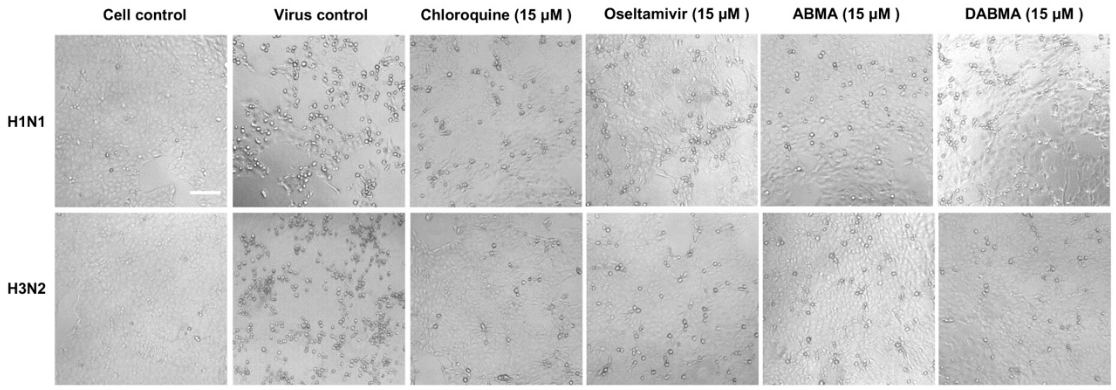

Figure S1. Protection effects of the indicated compounds against H1N1- and H3N2-induced CPE in MDCK cells. Morphological changes of MDCK cells were recorded at 48 h post A/NY/61/LV16A (H1N1) and A/17/HK/2014/8296 (H3N2) infection following treatment with the indicated compounds. Scale bar = 200 μm. Cells in the virus control group appeared scattered and detached compared with cells in the control group, while the cells in the compound treated groups were mostly round and spread out.

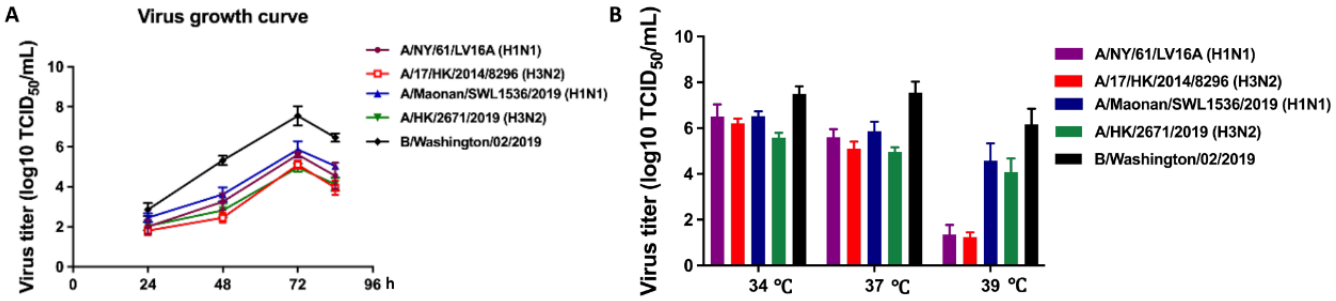

Figure S2. Growth of influenza virus strains in MDCK cells. (**A**) Influenza virus strain titers as a function of time. (**B**) Influenza virus strain titers as a function of temperature. Titers were measured by $TCID_{50}$.
